# ACKR3 promotes CXCL12/CXCR4-mediated cell-to-cell-induced lymphoma migration through LTB4 production

**DOI:** 10.3389/fimmu.2022.1067885

**Published:** 2023-01-12

**Authors:** Paola Antonello, Diego U. Pizzagalli, Mathilde Foglierini, Serena Melgrati, Egle Radice, Sylvia Thelen, Marcus Thelen

**Affiliations:** ^1^ Faculty of Biomedical Sciences, Institute for Research in Biomedicine, Università della Svizzera italiana, Bellinzona, Switzerland; ^2^ Graduate School of Cellular and Molecular Sciences, University of Bern, Bern, Switzerland; ^3^ Università della Svizzera italiana, Euler Institute, Lugano-Viganello, Switzerland; ^4^ Swiss Institute of Bioinformatics, Lausanne, Switzerland

**Keywords:** ACKR3, CXCR4, CXCR5, chemokine, atypical chemokine receptor, leukotriene B4, lymphoma characters

## Abstract

Chemotaxis is an essential physiological process, often harnessed by tumors for metastasis. CXCR4, its ligand CXCL12 and the atypical receptor ACKR3 are overexpressed in many human cancers. Interfering with this axis by *ACKR3* deletion impairs lymphoma cell migration towards CXCL12. Here, we propose a model of how ACKR3 controls the migration of the diffused large B-cell lymphoma VAL cells *in vitro* and *in vivo* in response to CXCL12. VAL cells expressing full-length ACKR3, but not a truncated version missing the C-terminus, can support the migration of VAL cells lacking ACKR3 (VAL-ko) when allowed to migrate together. This migration of VAL-ko cells is pertussis toxin-sensitive suggesting the involvement of a G_i_-protein coupled receptor. RNAseq analysis indicate the expression of chemotaxis-mediating LTB4 receptors in VAL cells. We found that LTB4 acts synergistically with CXCL12 in stimulating the migration of VAL cells. Pharmacologic or genetic inhibition of BLT_1_R markedly reduces chemotaxis towards CXCL12 suggesting that LTB4 enhances in a contact-independent manner the migration of lymphoma cells. The results unveil a novel mechanism of cell-to-cell-induced migration of lymphoma.

## Introduction

Cell migration is a widely conserved biological process, but the involved intracellular pathways are poorly understood. Directional migration or chemotaxis is orchestrated by distinct cell surface receptors, which guide cells along gradients of increasing concentrations of attractants (1). Chemokines, which constitute the largest family of chemoattractants, share a basic isoelectric point that allows them to be presented on cell surfaces and extracellular matrices (2, 3). They induce chemotaxis by binding to cognate G-protein-coupled receptors (GPCRs), which upon ligand binding activate *via* pertussis toxin-sensitive G proteins downstream pathways leading to cell migration (4, 5). In addition to conventional G-protein coupled chemokine receptors, chemokines bind to seven-transmembrane domain receptors, which do not activate G-proteins, called atypical chemokine receptors (ACKR) (3–5). ACKR3 acts mainly as a decoy with high affinity for the chemokine CXCL12 and with lower affinity for CXCL11, which are also ligands for the conventional receptors CXCR4 and CXCR3, respectively (6, 7). The physiological importance of the CXCL12/ACKR3/CXCR4 axis is underlined by the fact that absence of either component causes perinatal death in mice severely disrupting central nervous system and heart development (8–11).

Chemotaxis is governed by a chemokine-producing source, often constituted by stromal cells, and a juxtaposed sink that removes chemokines, either cells that express ACKRs or other mechanisms of degradation. Removal of chemoattractants is essential to maintain gradients and to avoid blurring of the source. Chemokine gradients are established over a relatively short distance of about 100 to 150 µm (12). Therefore, chemotaxis is not involved in long distance cell displacements as observed in passive blood-flow mediated cell movement (13).

In neoplasms, the chemokine system plays a promiscuous role depending on the type of cancer, the cellular and environmental context, and the involved chemokine axis. The chemokine system can influence disease progression in a beneficial or detrimental way (14–21). In many solid cancers altered expression levels of ACKR3 correlate with poor prognosis and tumor metastasis (22–29). In addition, the CXCL12/ACKR3/CXCR4 axis was proposed to be involved in lymphoma (30–32). Diffuse large B cell lymphoma (DLBCL) arise in the germinal center when B cells undergo somatic hypermutation and class-switch recombination. During these events, genetic alterations can cause malignant tumor transformation leading to GC-derived B cell lymphoma (33). We have previously shown that GC derived DLBCL VAL cells, require the functional expression of ACKR3 to disseminate into distant organs in NOD/SCID mice (32).

Tumor spreading is a multistage process where cancer cells undergo transformation, leave the primary tumor and migrate into adjacent and distant tissues, where they proliferate (34–38). It was previously assumed that cancers metastasize through single tumor cells escaping from the primary site arriving at distant tissues and propagating there. However, in the last decade, emerging evidence suggests that metastases can disseminate with a more complex mechanism called collective cell migration (39). This process describes the presence of leading cells that trace the way into adjacent tissues for the migration of follower cells (40) or induce the movement of cohesive clusters (39). Collective migration has been extensively studied to describe the metastasis of solid tumors but is seldom used to describe the movement of hematological cancers in a contact independent manner. Through chemokine sequestration expression of scavenger receptors can contribute to the formation of self-generated gradients (40, 41).

The mechanisms by which ACKR3, CXCR4 and CXCL12 induce the dissemination of VAL cells in a synergetic manner have not been addressed. Here, we demonstrate that in the presence of CXCL12 wild type VAL cells (VAL-wt), which can express ACKR3 on the cell surface, create cues that *in vitro* can be sensed by CXCR4^+^ follower VAL-wt. Moreover, VAL-wt support *in vitro* chemotaxis of VAL cells in which ACKR3 was genetically deleted (VAL-ko). The latter by themselves are not able to migrate in response to CXCL12. *In vivo* VAL-wt can induce the migration of CXCR4^+^ VAL-ko, contributing to the infiltration of draining lymph nodes. Using a novel computational analysis of 3D migration, we show that in the presence of CXCL12 VAL-wt stimulate cell-to-cell-induced migration presumably through the release of leukotriene B4 (LTB4). Moreover, the release of LTB4 from VAL cells induces cell polarization and chemotaxis in an autocrine and paracrine fashion. We propose a novel mechanism by which ACKR3 sustains the movement of otherwise migration incompetent cells and partially reveal the molecular components of this pathway.

## Results

### Chemokine receptor expression on DLBCL

**Figure 1:**
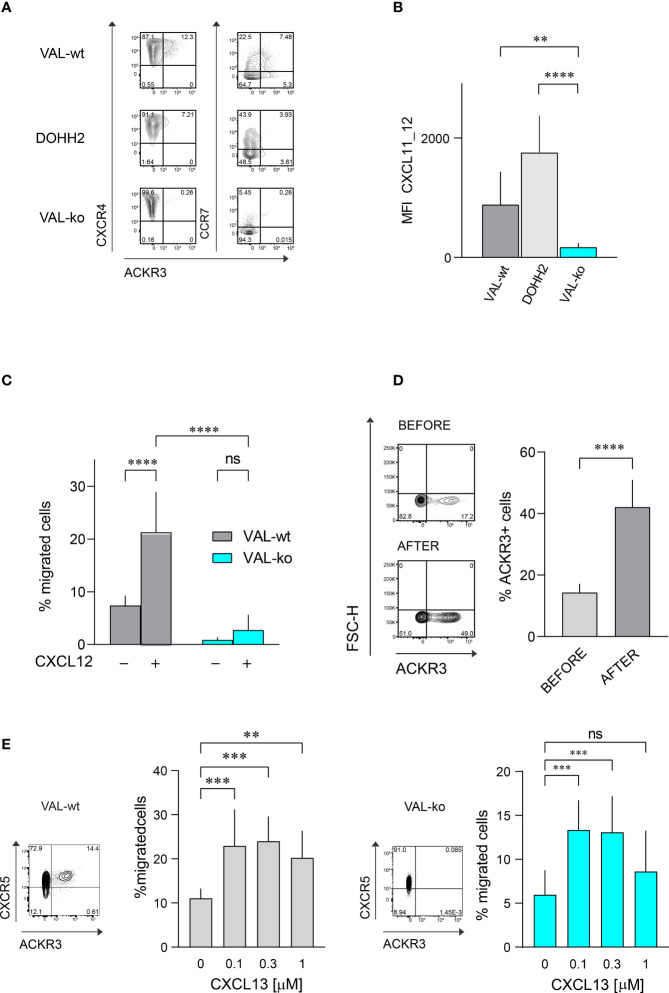
Chemokine receptor expression and function on DLBCL cells. **(A)** ACKR3 (mAb 11G8), CXCR4 (mAb 12G5) and CCR7 (mAb G043H7) surface expression on VAL-wt, DOHH2, and VAL-ko was measured by flow cytometry. Representative plots of three independent experiments. **(B)** Uptake of the chimeric chemokine CXCL11_12 AF647 by DLBCL. Mean fluorescence intensity (MFI) of DLBCL incubated with 50 nM of CXCL11_12 for 45 min at 37°C. MFI (by flow cytometry) reports the mean ± SD (Dunnett’s multiple comparisons, ONE-WAY ANOVA **p ≤ 0.01, ****p ≤ 0.0001) of four independent experiments performed in triplicates. **(C)** Transendothelial migration of lymphoma cells. VAL cell migration through mLEC towards 10 nM CXCL12 was measured after 8h incubation. Cell migration was calculated as percentage of the input (% migrated cells). Cumulative data from three independent experiments measured in triplicates (means ± SD, Turkey’s multiple comparison ONE-WAY ANOVA ns (not significant), p≥0.05, ****p ≤ 0.0001). **(D)** (Left) ACKR3 (mAb 11G8) surface expression on VAL-wt cells before (upper panel, before) and after transendothelial migration (lower panel, after) towards 10 nM CXCL12 as in **(C)**. Representative plots from ten independent experiments performed in duplicates. (Right) Percentage of cells expressing ACKR3 on the plasma membrane before and after migration. Cumulative data from five different experiments measured in triplicates (means ± SD, unpaired t-test, ****p ≤ 0.0001). **(E)** Left side: flow cytometry plot showing ACKR3 (mAb 11G8) and CXCR5 (mAb J252D4) surface expression on VAL-wt (left panel). Transendothelial migration of VAL-wt towards increasing concentrations of CXCL13 (0.1, 0.3 and 1 µM) (bar chart). Right side: flow cytometry plot showing ACKR3 (mAb 11G8) and CXCR5 (mAb J252D4) surface expression on VAL-ko (left panel). Transendothelial migration of VAL-ko through mLEC towards increasing concentrations of CXCL13 (0.1, 0.3 and 1 µM) (bar chart). Cell migration was calculated as described above. Bar charts show cumulative data of three independent experiments performed in triplicates (means ± SD, Dunnett’s multiple comparisons, ONE WAY ANOVA, **p ≤ 0.01, ***p ≤ 0.001).

The atypical chemokine receptor ACKR3 is known to be upregulated on both solid cancers and blood neoplasms (22, 24, 30, 31). Here we investigated its expression and functionality on two DLBCL lines, VAL and DOHH2. Flow cytometry was performed on wild type DLBCL cells and VAL cells in which ACKR3 was genetically ablated (VAL-ko). In the cells with deleted ACKR3 cyan fluorescent protein (CFP) was introduced under constitutively active spleen focus-forming virus (SFFV) promoter, which allows to easily identify cells in tissues and mixed cultures (32). We measured surface expression on these cells of ACKR3, CXCR4 and CCR7. All cells expressed similar levels of CXCR4 on the plasma membrane (**Figure 1A**). Conversely, ACKR3 was expressed only on a fraction of VAL-wt (10-18%), despite their clonal nature and continuous mRNA levels of the receptor (32). When cells were sorted for ACKR3 surface expression and cultured, the mixed phenotype reestablished rapidly within 2-3 weeks. However, cells negative for ACKR3 at sorting were more refractory for acquiring ACKR3 surface expression during this time (**Figure S1A**). However, during prolonged culture (6 weeks) the cell established the initial phenotype. Similarly, only a small fraction of DOHH2 expressed ACKR3 on the plasma membrane (**Figure 1A**). Interestingly, CCR7 was predominantly expressed on the surface of wild type DLBCL (VAL-wt and DOHH2), which were negative for ACKR3 plasma membrane expression. Genetic deletion of ACKR3 on VAL cells had no effect on CXCR4 expression. Surprisingly, CCR7 transcripts were less frequent in VAL-ko (117 ± 23 vs. 898 ± 32, (n=5)) and surface expression was markedly reduced on VAL-ko (**Figure 1A**).

To assess the functionality of ACKR3 we took advantage of a previously developed specific chemokine uptake assay (42). CXCL11_12 AF647 is a fluorescent chimeric chemokine side-specific labeled at the C-terminus, composed of the N-terminus of CXCL11 and the main body of CXCL12. The chimera specifically binds ACKR3, but not CXCR3 or CXCR4, the respective cognate receptors for CXCL11 and CXCL12. VAL-wt and DOHH2 readily internalized the chimera (**Figure 1B**). Hence, despite the limited surface expression of ACKR3 (**Figure 1A**), the receptor appears functional on both DLBCL lines. The observation is in agreement with the notion that the receptor cycles between the plasma membrane and endocytic compartments (6). As expected, VAL-ko did not scavenge the chimera and displayed low unspecific binding (**Figure 1B** and **Figure S1B**). Chemokine scavenging and receptor internalization requires the intact C-terminus of ACKR3 (6). We reintroduced into VAL-ko ACKR3 with a truncated C-terminus by stable transfection resulting in VAL-ACKR3-ΔC cells (VAL-ACKR3-ΔC). VAL-ACKR3-ΔC poorly scavenged CXCL11_12 AF647 indicating that the C-terminus of ACKR3 is required for scavenging and receptor internalization (**Figures S1B, C**), consistent with previous findings (6, 43).

Next, we assessed the chemotactic response towards CXCL12. Chemotaxis assays were performed as previously described with transwell plates coated with a monolayer of endothelial cells grown on a collagen-based extracellular matrix (32). The presence of endothelial cells optimizes the migration of lymphoma cells. VAL cells were allowed to migrate through a layer of mouse lymphatic endothelial cells (mLEC) for 8h in the absence and presence of an optimal concentration of CXCL12 (10 nM) in the lower compartment. In agreement with our previous findings (32), under these conditions about 20% of VAL-wt migrated into the lower compartment in the presence of chemokine, which accounts for a 3-fold increase compared to unstimulated chemokinesis. By contrast, VAL-ko, which express equal surface levels of CXCR4 showed no response to CXCL12, but also no spontaneous migration (**Figure 1C**). The observation was confirmed with genetically different clones to exclude off-target effects on cell migration through editing of ACKR3 by CRISPR/Cas9. Chemokinesis of VAL cells through mLEC was not inhibited in the presence of the CXCR4 antagonist AMD3100, suggesting a basal migratory activity that was not mediated by CXCR4 (**Figure S1D**). Similarly, VAL-ACKR3-ΔC, which express comparable CXCR4 levels on the surface (**Figure S1Ei**) did not perform CXCL12-dependent migration (**Figure S1Eii**). The observation suggests that the C-terminus is necessary for ACKR3 activity during VAL cell migration.

We next compared the surface expression of ACKR3 on VAL-wt before and after migration towards CXCL12. The fraction of cells, which expressed ACKR3 at the plasma membrane increased from about 17% to 40-50% after chemotaxis (**Figure 1D**). The observation could indicate that cells, which express ACKR3 at the plasma membrane, migrate more efficiently. However, it cannot be excluded that cells upregulate surface expression of the receptor during migration. To get a better insight we sorted ACKR3^+^ and ACKR3^-^ VAL cells and analyzed their migratory properties in chemotaxis assays. The efficiency of cells expressing ACKR3 at the surface was notably higher compared to cells that did not present ACKR3 at the plasma membrane. Moreover, VAL-wt lacking ACKR3 surface expression did not upregulate the receptor during migration (**Figure S1F**). Nevertheless, in ACKR3 negative VAL-wt the mRNA of the receptor is not absent compared to the genetically modified VAL-ko (32). Hence, it cannot be excluded that ACKR3 expressed on endosomal structures somehow contributes to the migration. All together, these findings suggest that functional ACKR3 plasma membrane expression is necessary to support efficiently CXCR4-dependent cell migration.

In order to exclude a general inhibitory effect on VAL cell migration by ACKR3 deletion we examined the chemotaxis towards CXCL13, the ligand for CXCR5, which is expressed on both, VAL-wt and VAL-ko (**Figure 1E**). With increasing concentrations of CXCL13 we observed a typical bell-shaped migration response by both cell types (**Figure 1E**), indicating that VAL-ko retain the capability to sense a chemokine gradient, polarize and migrate. In addition, we explored the ability of VAL-wt and VAL-ko to migrate in response to stimulation of CCR7 with CCL19. VAL-wt, which heterogeneously express CCR7 (**Figure 1A**), were sorted for CCR7 surface expression and tested separately. CCR7^+^ VAL-wt, which do not express ACKR3 on the surface (**Figure 1A**), migrated more efficiently than the CCR7^-^ ACKR3^+^ sorted cells (**Figure S1G**). The data indicate that the moderate chemotaxis towards CCL19 correlates with CCR7 surface expression. In line with this, VAL-ko cells, which do not express CCR7 (**Figure 1A**) did not migrate towards CCL19 (**Figure S1G**).

### VAL-wt promote migration of VAL-ko towards CXCL12

**Figure 2:**
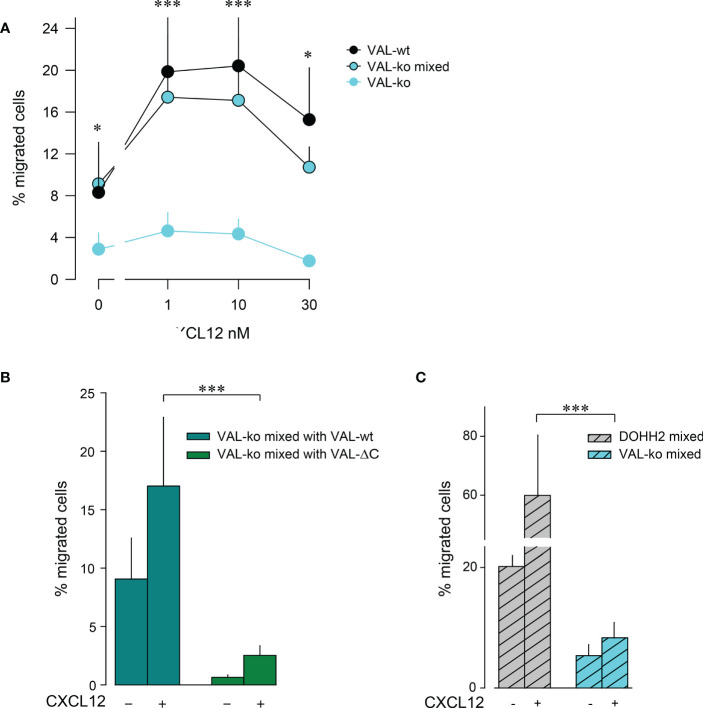
VAL-wt cells induce the migration of VAL-ko cells. **(A)** Transendothelial migration of VAL cells (as in **Figure 1**) towards different concentrations of CXCL12 (1, 10, 30 nM). Cyan symbols and cyan lines (VAL-ko) displays % migrated VAL-ko in the absence of VAL-wt. The % migrated VAL-ko (gated on CFP) in a 1:1 mixture with VAL-wt was calculated from the input of VAL-ko in the mixture (VAL-ko mixed, cyan filled symbols and black lines). Black symbols: % migrated VAL-wt calculated from the respective input. Calculation by flow cytometry as in **Figure 1**. Cumulative data of three independent experiments measured in triplicates (means ± SD, Dunn’s multiple comparisons ONE WAY ANOVA, *p ≤ 0.05, ***p ≤ 0.001) **(B)** Transendothelial migration of VAL cells towards 10 nM CXCL12 or medium. VAL-ko were mixed 1:1 with VAL-wt (dark cyan) or VAL-ΔC (green) and the % migrated VAL-ko was calculated from the input of VAL-ko in the mixture. Quantification was performed as described in **Figure 1**. Cumulative data of three independent experiments measured in triplicates (means ± SD, Turkey’s multiple comparison, ONE WAY ANOVA, ***p ≤ 0.001). **(C)** Migration of DOHH2 cells. DOHH2 cells were mixed 1:1 with VAL-ko and the mixture allowed to migrate for 8 h towards 10 nM CXCL12 or medium. The % migrated DOHH2 (grey bars) and of VAL-ko (cyan bars) were calculated from the respective inputs in the mixture. Experiments performed in triplicates, representative example of three independent experiments (means ± SD, Turkey’s multiple comparison, ONE WAY ANOVA ***p ≤ 0.001).

The observation that VAL-wt, which do not express ACKR3 on the surface, can migrate towards CXCL12 in the presence of cells exposing the receptor on the plasma membrane was intriguing (**Figure 1D**). Previous data showed that in zebrafish ACKR3 expressed *in trans* on stromal cells can support the CXCL12-mediated migration of primordial germ cells (44). It is plausible that ACKR3 positive VAL-wt, when locally scavenging CXCL12, shape micro-gradients that are sensed by CXCR4^+^ VAL cells that do not express ACKR3 on the surface. The hypothesis implies that ACKR3 scavenging activity is necessary for VAL cell migration. To address the assumption, we used mLEC transwell assays to measure CXCL12-induced chemotaxis of VAL-ko in the presence of VAL-wt. We mixed VAL-wt and CFP^+^ VAL-ko in a 1:1 ratio, which enables the identification of the two cell types by flow cytometry. The mixture was allowed to migrate towards different concentrations of CXCL12 (**Figure 2A**). CFP^+^ VAL-ko in the 1:1 mixture with VAL-wt showed a typical bell-shaped migratory response towards CXCL12, whereas VAL-ko cells alone poorly responded (**Figure 2A**). Moreover, in the mixture, the CFP^+^ VAL-ko cells migrated with a similar efficiency as VAL-wt cells (**Figures 2A, B**). Next, we addressed the role of the C-terminus of ACKR3 in promoting the migration of VAL-ko. VAL-ACKR3-ΔC, barely supported VAL-ko migration towards CXCL12 in a 1:1 mixture, highlighting the importance the C-terminus for scavenging (**Figure S1C**), migration (**Figure S1Eii**) and stimulation *in trans* (**Figure 2B**). It is conceivable that the scavenging activity is essential for chemotaxis of ACKR3 expressing cells and to support migration of VAL-ko, however it cannot be excluded that the C-terminus mediates additional intracellular signaling unrelated to receptor trafficking. The later assumption is in line with previous findings (9) where it was shown that phosphorylation-deficient (C-terminus) ACKR3 does not scavenge chemokines but rescues the lethal phenotype of receptor-deficient mice.

To extend the observation of DLBCL migration we measured mLEC transwell chemotaxis of DOHH2 cells in response to CXCL12. DOHH2 cells migrated more efficiently than VAL-wt, but also displayed enhanced chemokinesis. When DOHH2 cells were mixed with VAL-ko cells they induced chemokinesis of VAL-ko cells and barely supported chemotaxis towards CXCL12 (**Figure 2C**). Taken together, the data suggest that expression of full-length ACKR3 on leader cells is needed to induce the CXCL12-dependent cell-to-cell-induced migration of CXCR4^+^ VAL-ko.

### Migration of VAL-ko from the tumor to the draining lymph node requires the presence of VAL-wt or DOHH2 cells

**Figure 3:**
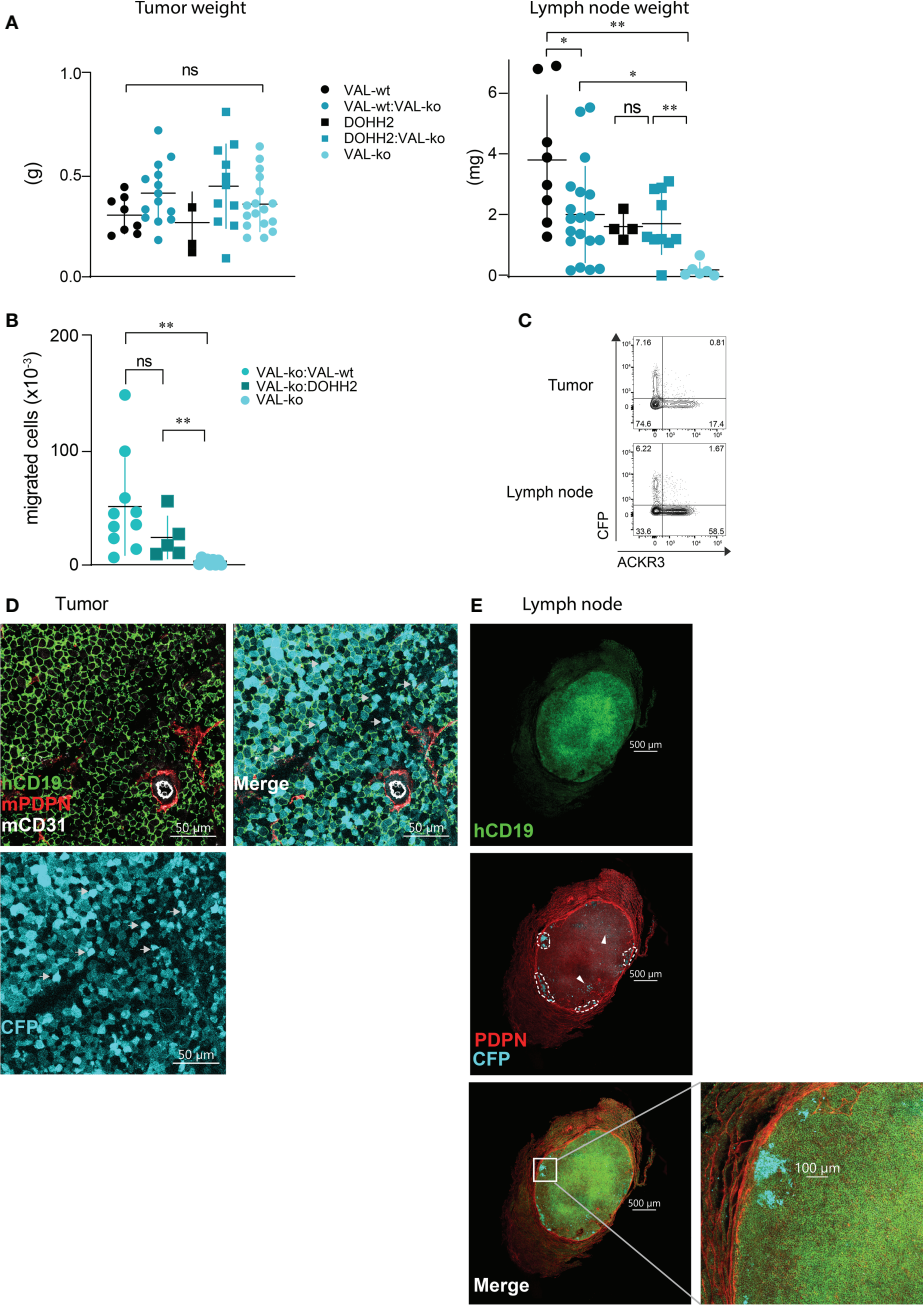
Migration of VAL-ko from the primary tumor to the draining lymph node is enhanced in the presence of ACKR3 expressing cells. **(A)** Tumor (left) and lymph node (right) weights from NOD/SCID common γ-chain^ko^ mice injected with 10^7^ VAL-wt, VAL-ko and DOHH2 cells alone and as 1:1 mixture VAL-wt:VAL-ko and DOHH2:VAL-ko. Weights are reported as means ± SD from three independent xenograft experiments with 4-5 animals per group. Tumor and lymph node t-test single comparison: ns, p≥0.05, *p≤ 0.05, **p ≤ 0.01. **(B)** Total number of CFP^+^ VAL-ko present in the draining axillary lymph node from the same experiments. Lymph node cells were analyzed by flow cytometry and gated for hCD19^+^ (HIB19 or SJ25C1), mCD45^-^ (30-F11) and CFP^+^ expression (means ± SD, t-test single comparison, ns, p≥0.05 **P ≤ 0.01). **(C)** Surface expression of ACKR3 on VAL-wt cells and CFP expression in VAL-ko extracted from a representative localized tumor (upper panel) and from the corresponding lymph node (lower panel). Representative data from one experiment shown in A (n= 4-5). **(D)** Confocal images of sections of a tumor generated by injecting a 1:1 mixture of VAL-wt and VAL-ko (CFP^+^). Tumors were fixed for 48h in a solution of 4% paraformaldehyde and Vibratome sections stained overnight with anti-hCD19 (mAb HIB19) anti-mPDPN (mAb 8.1.1) and anti-mCD31 (mAb MEC13.3). **(E)** Confocal images of sections of a draining lymph node from a tumor generated by injecting a 1:1 mixture of VAL-wt and VAL-ko (CFP^+^). Lymph nodes were fixed for 5h in a solution 4% paraformaldehyde and Vibratome sections were stained overnight with anti-hCD19 Ab (mAb HIB19), anti-mPDPN (mAb 8.1.1).

We used a previously reported localized tumor xenograft model in NOD/SCID mice (32) to investigate lymphoma cell migration *in vivo*. We injected 10^7^ lymphoma cells subcutaneously in the flanks of NOD/SCID mice. All animals showed a comparable increase in body weight and tumor size (**Figure 2SA**). After 20-21 days, animals were sacrificed and the mass of the primary tumor was measured. No significant differences in mass were observed between tumors grown from wild type DLBCL, VAL-wt cells and DOHH2, VAL-ko and the respective mixtures of wild type and VAL-ko (**Figure 3A**). NOD/SCID mice barely developed sizable lymph nodes due to the lack of endogenous lymphocytes. Injection of wild type DLBCL cells led to measurable mass of the draining lymph node. By contrast, the draining lymph nodes of tumors from VAL-ko remained hardly detectable (**Figure 3A**). However, an increase in lymph node mass was observed, when 1:1 mixtures of wild type DLBCL and VAL-ko were injected (**Figure 3A**). To test whether under these conditions VAL-ko contributed to the increase in mass of the draining lymph node, we used flow cytometry to measure the presence of CFP^+^ VAL-ko. While VAL-ko alone did not migrate to the axillary lymph node, in the presence of VAL-wt or DOHH2 the CFP^+^ VAL-ko could readily be detected (**Figure 3B**). Hence, like *in vitro*, also *in vivo* wild type lymphoma cells appeared to promote the migration of ACKR3 deficient VAL-ko. In the mixture preferentially ACKR3 positive VAL-wt migrated to the draining lymph node along with VAL-wt, which do not express ACKR3 at the surface and CFP^+^ VAL-ko (**Figure 3C**). Consistent with our *in vitro* observations (**Figure 1D**) the proportion of VAL-wt expressing ACKR3 on the surface was markedly increased from 18% to over 50% in the draining lymph node. By contrast, the expression of CCR7 was unaltered on VAL-wt and remained essentially absent on CFP^+^ VAL-ko, suggesting that the receptor may not contribute to draining lymph node infiltration (**Figure S2B**).

Immunofluorescence (IF) analysis of sections from tumors of VAL-wt:VAL-ko mixtures by confocal microscopy revealed that VAL-wt and VAL-ko are randomly distributed in the tumor mass. Using podoplanin (PDPN) and CD31 staining for mouse lymphatic and endothelial vessels we found a similar localization for both cell populations (**Figure 3D**). By contrast, IF analysis of axillary lymph node sections from mice bearing VAL-wt:Val-ko tumors revealed striking differences in the distribution of the two cell populations. Human CD19^+^ VAL-wt almost completely populated the entire draining lymph node. By contrast, CFP^+^ VAL-ko were found at the edge of the lymph node, close to the sub-capsular sinus in clusters surrounded by VAL-wt cells (**Figure 3E**). Taken together, the data indicate that like *in vitro* lymphoma cells can also promote the migration of VAL-ko *in vivo.*

### Computational analysis reveals cell-to-cell-induced 3D migration

**Figure 4:**
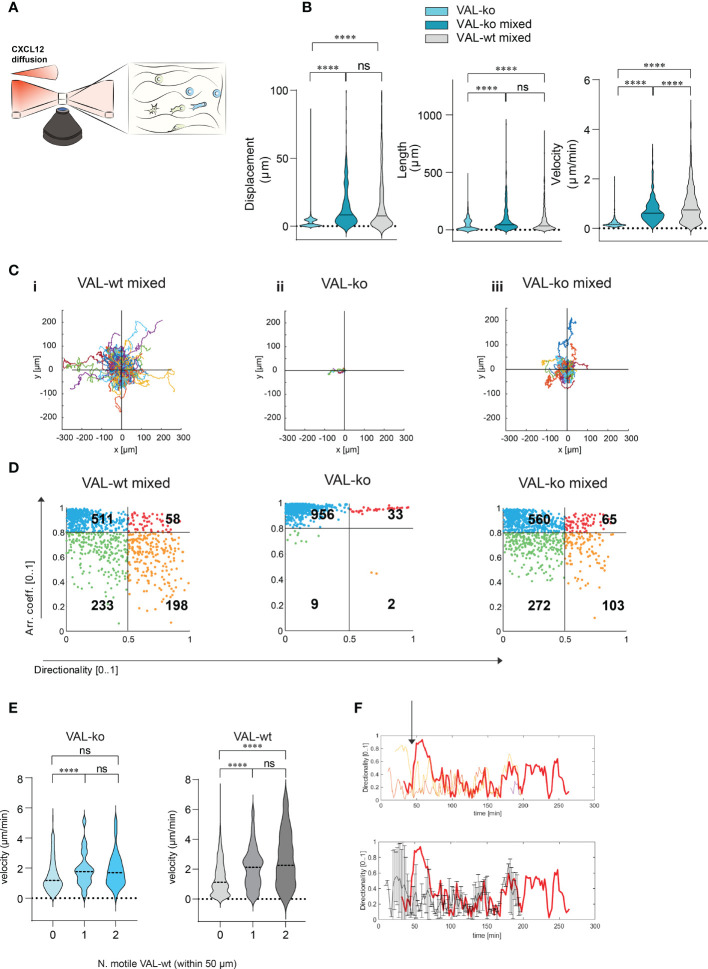
CXCL12 stimulated VAL-wt increase the 3D motility and directional migration of VAL-ko. **(A)** Schematic representation of 3D migration chamber showing a chemotactic gradient (CXCL12 diffusion) from left to right. **(B)** Violin plots showing displacement, length and velocity of VAL-ko alone (light cyan), VAL-ko in a 1:1 mixture with VAL-wt (VAL-ko mixed, dark cyan) and VAL-wt in the same mixture (VAL-wt mixed, grey). Tracking analyses were performed using Imaris software (means, Turkey’s multiple comparisons test, ONE-WAY ANOVA ns, p≥0.05, ****p ≤ 0.0001). **(C)** Plots of tracks with a common origin. (i) VAL-wt mixed, (ii) VAL-ko alone, (iii) VAL-ko mixed. **(D)** Scatter plots of directionality *vs* arrest coefficients of 1000 VAL-wt mixed, VAL-ko alone, VAL-ko mixed. Gates with differently colored dots indicate four different motility patterns. **(E)** Violin plots of changes in velocity of VAL-ko and VAL-wt, relative to the number of motile (0, 1, 2) VAL-wt in a 50 µm radius (means, Mann-Whitney t-test ns, p≥0.05, ****p ≤ 0.0001). **(F)** Directionality changes over time of a VAL-ko (red thick line) with respect to directionality changes of two VAL-wt in a 50 µm radius (orange and red thin lines, upper plot). The lower plot shows the same directionality changes of a single VAL-ko (red thick line), compared to the average of directionality value of VAL cells in the 50 µm radius.

To investigate further the migration and the cooperation between cells, we used a 3D migration and quantitative computational (45) analysis system. Cells were embedded in a collagen matrix and chemotaxis towards CXCL12 was followed by time-lapse imaging (**Figure 4A**). Tracks of migrating cells were generated automatically by computational analysis and used to extrapolate data of statistical significance to calculate displacement, track length and velocity of single cells. Single cell data were pooled into experimental groups for comparison (**Figure 4B**). The analysis confirmed that VAL-ko are unable to migrate towards CXCL12 (**Figure S3A**) while the mixture of migrating VAL-ko:VAL-wt is mobile and migration competent (**Figure S3B**). Displacement and track length of VAL-ko in the mixture is comparable to that of VAL-wt (**Figure 4B**). However, cells in the mixture appeared to migrate with an intermediate velocity being faster than VAL-ko cells alone, but slower than VAL-wt cells (**Figure 4B**). Similarly, plotting the tracks on common origin revealed that (i) VAL-wt in the mixture are highly motile, (ii) VAL-ko essentially do not move, while (iii) the migratory capacity of VAL-ko is partially restored in the presence of VAL-wt (**Figure 4C**). The results are consistent with the transwell assays and the *in vivo* data, demonstrating that the presence of ACKR3 is essential to promote migration *in cis* and *in trans*.

To better characterize differences in motility, we analyzed the behavior of individual cells over time. We applied an algorithm using mobility values to generate plots that distinguish four populations according to their migratory behavior. We quantified instantaneous motility measures over time, by dividing each track into fragments (tracklets), and performed a tracklet-based action analysis as previously described (46). From the motility measures, we computed the continuous arrest coefficient (46), that is the proportion of time during which a cell does not move. Cells with low motility are associated with high values of arrest coefficient. Moreover, we computed the instantaneous directionality (46), which was associated with high values for polarized cells migrating along straight trajectories. We assigned these coefficients to 1000 tracklets of each group: VAL-wt and VAL-ko in the mixture (separately analyzing CFP^-^ and CFP^+^ cells) and VAL-ko alone (**Figure 4D**). For VAL-wt, in a 1:1 mixture with VAL-ko, 233 tracklets displayed low arrest coefficients and low directionality, which indicates that these cells perform chemokinesis (green gate), whereas 511 tracklets were associated with immobile cells, characterized by tracklets with a high arrest coefficient and low directionality (blue gate). Tracklets of cells, which protruded lamellipods, hence visibly polarized, but showed slow or no migration (red gate 58 tracklets), were defined by a high arrest coefficient and high directionality (deduced from the polarity). Finally, 198 tracklets (orange gate) displayed low arrest coefficients and high directionality and were associated with cells that performed a pronounced directional migration. VAL-ko when placed alone in the chamber were found to be mostly immobile (956 tracklets in the blue gate). However, VAL-ko in the mixture displayed similar motility as VAL-wt with slightly less directional migration ability (103 vs. 198 tracklets). Our algorithm applied to a large number of tracklets confirmed the motility of VAL-wt and their ability to promote the migration of VAL-ko.

We further analyzed whether the velocity of VAL-ko and VAL-wt migration depends on a motile VAL-wt in the direct neighborhood. **Figure 4E** shows that a VAL-ko in the presence of at least one motile VAL-wt within a radius of 50 µm migrated with significantly higher velocity compared to VAL-ko where no motile VAL-wt were in close proximity. We observed that the increase in velocity of migration of VAL-ko was similar whether one or two motile VAL-wt were in the neighborhood, suggesting no additive effect. The same analysis performed for the velocity of VAL-wt revealed that the motility of the cells was also influenced by the presence of a motile VAL-wt within a radius of 50 µm. The finding suggested that VAL-ko and VAL-wt might perform a secondary migration, which could be triggered either by microgradients of CXCL12 created by ACKR3^+^ VAL-wt or by another stimulus that is released during the migration of VAL-wt along a CXCL12 gradient.

By analyzing in depth the tracks of VAL-ko we saw that the cells often migrate to a spot where a VAL-wt had briefly paused and left (Movies S1-3). In addition, we measured the changes in directionality of VAL-ko and VAL-wt in close proximity and plotted the data over time (**Figure 4F**). We found that the changes in directionality of VAL-ko were in most cases preceded by a change in VAL-wt directionality (**Figure 4F** upper panel). Moreover, the migration of a VAL-ko was similar to the overall movement of motile cells in a radius of 50 µm (**Figure 4F**, lower panel). The observations support the assumption that the migration of VAL-ko depends on the movement of VAL-wt, in line with the hypothesis that DLBCL invade tissues through cell-to-cell-induced migration as a heterogeneous population of cells.

### Pertussis toxin treatment of VAL-wt inhibits the cell-to-cell-induced migration of VAL-ko

**Figure 5:**
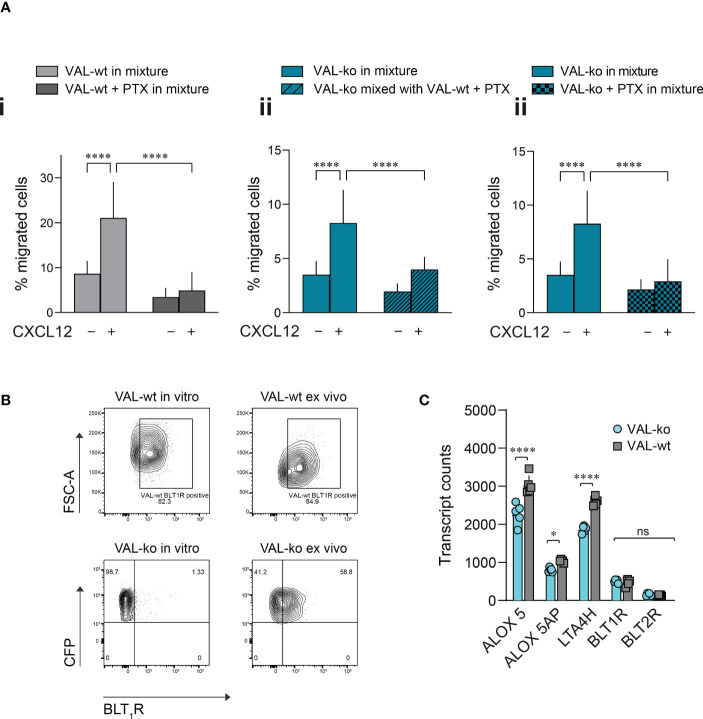
VAL-ko migration in the presence of VAL-wt is PTX sensitive. **(A)** Transmigration towards 10 nM CXCL12 was performed and calculated as described in **Figure 1**. (i) VAL-wt untreated (light grey) or treated with PTX (dark grey) in the mixture with untreated VAL-ko. (ii) VAL-ko mixed with untreated (left, plain bars) or PTX-treated VAL-wt (right, hatched bars). (iii) VAL-ko untreated (left, plain bars) or PTX-treated (right, hatched bars) mixed with untreated VAL-wt. Cumulative data of three independent experiments performed in triplicates (means ± SD, Turkey’s multiple comparisons test, ONE WAY ANOVA, ****p ≤ 0.0001). **(B)** BLT1R (mAb 203/14F11) surface expression on VAL-wt (upper panels) and VAL-ko (lower panel) cultured *in vitro* (left panels) or *ex vivo* (right panels). Representative plots of three different flow cytometry analyses. **(C)** Transcript levels of the lipoxygenases ALOX5 and ALOX5AP, the hydrolase LTA4H and the LTB4 receptors BLT1R and BLT2R of VAL-wt and VAL-ko (means ± SD, Šídák multiple comparison, 2way ANOVA ns p≥0.05, *p ≤ 0.05, ****p ≤ 0.0001) ns (not significant).

Pertussis toxin (PTX) treatment inhibits the transmigration of VAL-wt towards CXCL12 but has no effect on the VAL cell chemokinesis (**Figure S4A**). We next mixed PTX-pretreated VAL-wt in a 1:1 ratio with untreated VAL-ko and subjected them to transmigration assays towards CXCL12. As expected, inhibition of G_i_PCRs in VAL-wt abolished their migration towards CXCL12 in the mixture with VAL-ko (**Figure 5Ai**). Moreover, pretreatment of VAL-wt cells with PTX significantly reduced their ability to promote the migration of untreated VAL-ko cells (**Figure 5Aii**). To investigate the possible involvement of a G_i_-protein coupled chemotactic receptor in the migration of VAL-ko cells, we preincubated VAL-ko with pertussis toxin and mixed them with untreated VAL-wt. Preincubation of VAL-ko with PTX inhibited their migration (**Figure 5Aiii**), suggesting that the migration of VAL-ko in the presence of VAL-wt depends on the activation of a chemotactic G_i/o_-protein coupled receptor.

To identify such chemotactic receptor, we performed RNA-seq analyses of the transcriptomes of VAL-wt and VAL-ko. Comparing the data revealed that in general the expression levels of GPCRs transcripts showed no gross differences between the two cell types (**Figure S4B**). Of the known chemotactic receptors, the transcripts of CCR7 were higher expressed in VAL-wt (log_2_ fold 2.9) in agreement with our FACS data (**Figure 1A**, **Figure S4B**). GPR183, also known as EBI2, a chemotactic receptor for dihydroxysterols (47–49) was markedly higher expressed in VAL-wt (log_2_ fold 4.1) (**Figure S4B**). However, we could not observe a chemotactic response of VAL cells towards dihydroxysterols (not shown). Both cell types expressed similar transcript levels of CXCR5 and CXCR4 (**Figure S4B**) in agreement with the flow cytometry data and the chemotaxis experiments. In addition, we found similar levels of transcripts for two G_i_-protein coupled receptors for leukotriene B4 (LTB4), namely BLT_1_R (BLT1R) and BLT_2_R (BLT2R) (**Figure S4B**), although the transcripts of both receptors were less frequent than those of chemokine receptors. The transcript levels of both receptors were similar in VAL-wt and VAL-ko, however BLT_2_R transcripts were slightly higher in both cell types. BLT_1_R is well known to mediate chemotaxis of leukocytes in response to LTB4 (50), while the ubiquitously expressed BLT_2_R is more involved in regulating inflammatory responses, rather than cell migration (51, 52). We therefore measured the surface expression of BLT_1_R on VAL-wt and VAL-ko by flow cytometry. Despite similar transcript levels, VAL-wt expressed higher levels of BLT_1_R than VAL-ko in *in vitro* culture (**Figure 5B**). Interestingly, VAL-ko extracted from local tumors of NOD/SCID mice, showed a marked increase of surface BLT_1_R surface expression (up to 60% positive cells) (**Figure 5B**), suggesting that the tumor microenvironment promoted the upregulation of surface BLT_1_R, either from intracellular stores or *via de novo* translation. VAL-wt *ex vivo* did not display changes at surface expression of BLT_1_R compared to VAL-wt cultured *in vitro* (**Figure 5B**). The transcriptome analysis indicated further that several critical enzymes of the LTB4 synthesis pathway, such as the hydrolase LTA4H, the lipoxygenases ALOX5 and ALOX5AP were significantly more frequent in VAL-wt compared to VAL-ko (**Figure 5C**). We also sorted DOHH2 cells for ACKR3 surface expression and measured the transcriptomes. Both populations (ACKR^+^ *vs* ACKR^-^) expressed comparable levels of the transcripts for BLT_1_R (424.6 ± 66 *vs* 332 ± 56.5), LTA4H (3012 ± 102.7 *vs* 2894 ± 208.5), ALOX5 (1870 ± 154 *vs* 1819 ± 237) and ALOX5AP (688.2 ± 154 *vs* 708.1 ± 236) like VAL-wt.

### CXCL12 and LTB4 synergistically induce the migration of DLBCL

**Figure 6:**
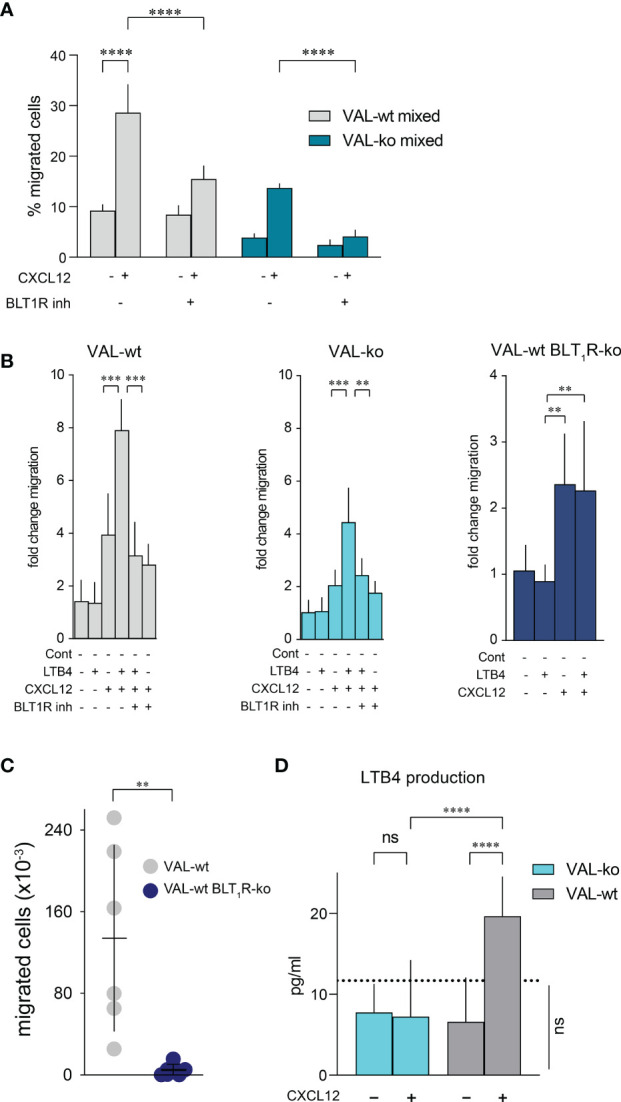
CXCL12 and LTB4 synergistically induce the migration of DLBCL. **(A)** Transmigration through mLEC was performed and calculated as described in **Figure 1**. Migration towards 10 nM CXCL12 of VAL-wt in a 1:1 mixture with VAL-ko (VAL-wt mixed, grey bars) and VAL-ko in a 1:1 mixture with VAL-wt (VAL-ko mixed, dark cyan bars) was measured in the absence and presence of 1 µM of the BLT1R inhibitor, BIIL315. Cumulative data of three independent experiments performed in triplicates (means ± SD, ONE WAY ANOVA, Turkey’s multiple comparisons test, ****p ≤ 0.0001). **(B)** VAL cells were embedded in a collagen matrix on the transwell insert. Fold change of VAL-wt (left, grey bars), VAL-ko (middle, cyan bars) and VAL-wt with deleted *blt_1_r* gene (right, blue bars) migrating in response to 10nM LTB4 and 10 nM CXCL12 in the absence and presence of 1 µM BLT1R inhibitor (BIIL315) as indicated. Cumulative data from three experiments performed in quadruplicates (means ± SD, Student’s t-test **p ≤ 0.01 ***p ≤ 0.001). **(C)** Total number of VAL-wt (gray dots) and VAL-wt lacking BLT_1_R (dark blue dots) present in the draining axillary lymph node. Lymph node cells were analyzed by flow cytometry and gated for hCD19^+^ (HIB19 or SJ25C1) and mCD45^-^ (30-F11) expression (means ± SD, t-test single comparison, **p ≤ 0.01). **(D)** LTB4 production (pg/mL) by VAL-wt and VAL-ko in absence or presence of 10 nM CXCL12. Values below the dashed line are under the sensitivity level. Cumulative data of three independent experiments performed in duplicates or quadruplicates (mean ± SD, ONE WAY ANOVA, Turkey’s multiple comparison test, ****p ≤ 0.0001).

The expression of enzymes involved in LTB4 production suggested that CXCL12 and LTB4 could act synergistically in stimulating VAL-wt migration through a positive feedback loop. Moreover, it could explain why cells, which are in the proximity of a LTB4 secreting cell, respond more efficiently to CXCL12 (**Figure 4E**). To test the possible synergistic involvement of the BLT_1_R in cell-to-cell-induced migration of VAL cells, we measured transwell chemotaxis of VAL-wt and VAL-ko towards CXCL12 in the presence of the BLT_1_R inhibitor, BIIL315. BIIL315 markedly attenuated CXCL12-stimulated migration of VAL-wt. Moreover, the inhibitor abolished VAL-wt-mediated migration of VAL-ko in response to CXCL12 (**Figure 6A**). The observation suggested the involvement of the LTB4/BLT_1_R axis in the cell-to-cell-induced migration of VAL cells. However, the reduced migration of VAL-ko could also result from the lower number of VAL-wt migrating in the mixture. To measure LTB4-stimulated chemotaxis, we embedded VAL cells in a collagen matrix coating the transwell to exclude metabolic interference of an endothelial cell layer. In general, under these conditions chemotaxis of VAL cells was less efficient. We observed that LTB4 alone did not induce migration of VAL cells (**Figure 6B**). However, CXCL12- stimulated chemotaxis was markedly enhanced in the presence of LTB4. Similarly, CXCL12-induced migration of DOHH2 cells was markedly enhanced in the presence of LTB4 (**Figure S4C**). The synergistic effect of LTB4 was impaired in the presence of the BIIL315 confirming the involvement of BLT_1_R (**Figure 6B**).

To further confirm the synergism between BLT_1_R and CXCR4 signaling we deleted the *blt_1_r* gene on all alleles in VAL-wt using CRISPR/Cas9 technology (**Figure S4D**) and measured transwell migration in response to CXCL12 and LTB4. Deletion of the *blt_1_r* gene completely abrogated the LTB4-mediated enhancement of CXCL12-stimulated migration (**Figure 6B**). By contrast, CXCR13-stimulated migration of VAL-wt, VAL-ko and VAL-wt with deletion of *blt_1_r* was not enhanced in the altered of LTB4 (**Figure S4E**). Together the observations indicate a synergistic effect of LTB4 on CXCR4/CXCL12- mediated VAL cell migration *in vitro* (**Figure 6B**). To confirm the relevance of the finding *in vivo* we injected VAL-wt and VAL-wt BLT_1_R ko (cells lacking BLT_1_R) into the flanks of mice. Tumors from both cell lines grew equally and had similar weights on the day of sacrifice (**Figure S4F**). However, while the draining axillary lymph nodes were readily detected in animals injected with VAL-wt, these were essentially absent when mice were inoculated with VAL-wt BLT_1_R ko cells (**Figure 6C**). The results are in line with the *in vitro* observations indicating that LTB4/BLT_1_R signaling is required for cell-to-cell-induced migration.

We next measured the release of LTB4 by VAL-wt and VAL-ko cells. Stimulation of CXCR4 with CXCL12 leads to the PTX-sensitive activation of phosphoinositide lipase C and *via* phospholipase A_2_ to the formation of arachidonic acid, a critical precursor of LTB4. Inhibiting 5-lipoxigenase with Demethylnobiletin blocks LTB4 formation (53). In line with the induction of cell-to-cell-induced migration, CXCL12 stimulated in VAL-wt a significant over background (~ 10 pg/mL) release of LTB4 (**Figure 6D**). By contrast, LTB4 production was not observed in VAL-ko and was fully inhibited by treatments with PTX or Demethylnobiletin (data not shown).

## Discussion

We demonstrate here that CXCR4-mediated migration of lymphoma cells towards CXCL12 requires the expression of functional ACKR3 at the cell surface. The conclusion is further supported by the finding that VAL-wt, which express ACKR3 at the surface, enhance the migration of cells that do not express ACKR3 at the plasma membrane, but contain the transcripts for the receptor, a finding that is reproducible both *in vitro* and *in vivo*. Moreover, VAL-wt can induce CXCL12-dependent chemotaxis of otherwise migration-deficient VAL-ko *in vitro*. The same stimulatory effect of ACKR3^+^ VAL cells is also observed *in vivo*, where VAL-wt promote lymph node infiltration of VAL-ko. These observations indicate that ACKR3 can act in *trans*, not necessarily being expressed on the same cell as the chemotaxis mediating CXCR4. Computer-assisted quantitative analysis of 3D migration hinted the presence of a secondary chemotactic cue created by VAL-wt, which induces the migration of VAL-ko. RNAseq data revealed the presence of transcripts for the expression of the chemotactic receptor BLT1R. In fact, we demonstrate that the BLT1R ligand LTB4 supports CXCR4-mediated chemotaxis acting synergistically with CXCL12 in triggering VAL cell chemotaxis. Moreover, upon stimulation with CXCL12, VAL-wt secrete LTB4, hence creating a positive feedback loop for contact-independent cell-to-cell-induced migration.

ACKR3 surface expression is heterogeneous on VAL cells. In culture the majority of cells do not express the receptor on the plasma membrane, despite the presence of similar levels of transcripts in receptor positive and negative cells. It is plausible that in ACKR3^-^ cells the receptor may reside in endosomes. In order to support CXCR4-mediated migration, however, ACKR3 has to be expressed on the cell surface. Removing the C-terminus of ACKR3 causes its plasma membrane localization on all cells, but the truncation renders the receptor incapable to enhance CXCR4-dependent chemotaxis. The finding suggests that the C-terminus is not only involved in receptor localization, trafficking and chemokine scavenging, but is also part of a signaling cascade leading to LTB4 production. Genetic ablation of ACKR3 in VAL cells almost completely abrogates their chemotactic response to CXCL12, albeit continuous expression of CXCR4. The inhibition of CXCR4/CXCL12 signaling in VAL-ko was not observed when chemotaxis was stimulated *via* the CXCL13/CXCR5 axis, indicating a selective interference of ACKR3 in the CXCR4 signaling pathway.

Heterodimerization of chemokine receptors has been suggested to alter their respective activities (54–57). However, functional interaction of chemokine receptors may depend on the cellular context. It was reported that CCR7 inactivates CXCR4 (56) while in another report CXCR4 expression was shown to be required for stable CCR7 expression (58). Our observation that CCR7 is downregulated in VAL-ko despite unaltered CXCR4 expression may indicates that ACKR3 is involved in CCR7 expression. On the other side it has been proposed that ACKR3 can form heterodimers on the plasma membrane with CXCR4 altering its activity (8, 59). We demonstrate here that ACKR3 expression in *trans* is sufficient to support CXCR4-mediated chemotaxis of VAL-ko *in vitro* and *in vivo*. Furthermore, our 3D time-lapse migration analysis strongly supports the view that the CXCR4-stimulated migration of ACKR3-deficient cells is contact independent. Interestingly, in many instances, regardless of ACKR3 expression, cells arrest at spots where a leader cell stopped before and typically followed the direction. The data further support a mechanism of cell-to-cell-induced migration that is contact-independent. In line with these observations is the finding that cells in a radius of 50 µm are sufficient to enhance the migration of slow-moving cells. Our data suggest that LTB4 could be the mediator of the contact-independent enhancement of CXCL12-stimulated migration of ACKR3-deficent cells. In fact, LTB4 synergistically enhances CXCL12-mediated migration of VAL cells and pharmacologic inhibition of BLT_1_R or genetic abrogation of the receptor reverts the effect. The assumption is in agreement with reports showing that B-cells can secrete LTB4 (60, 61) and with studies showing that neutrophils secrete LTB4 to induce swarming. Nevertheless, the LTB4-mediated migration reported here differs from neutrophil swarming over long distances since the effect of LTB4 appears to be confined to less than 50 µm (62, 63). Moreover, it was reported that LTB4 upregulates chemotactic receptors in leukocytes promoting chemotaxis toward their respective ligands (64), confirming the presence of a positive feed-back between LTB4 release and chemotaxis. The involvement of LTB4 in the recruitment of neutrophils in combination with other chemoattractants to inflamed joints was also shown (65, 66). Our data suggest that the synergistic effect of LTB4 and CXCL12 is specific for CXCR4-induced chemotaxis since LTB4 has no effect on CXCR5-mediated migration of VAL-wt. Nevertheless, we cannot fully exclude that CXCL12 scavenging by ACKR3 creates microgradients, which somehow modify CXCR4/CXCL12 signaling on ACKR3 negative cells. Further studies are required to describe the molecular mechanism that supports the release of LTB4 upon ACKR3/CXCR4 stimulation.

Using a diffuse xenograft model we showed that spreading of VAL cells into tissues, such as brain, bone marrow and spleen, requires ACKR3 expression (32). Given the poor prognosis of DLBCL patients with brain infiltrations (67, 68), understanding the underlying mechanism is of great clinical relevance. Here we report that ACKR3 is required for DLBCL to escape from local tumors to move to draining lymph nodes resembling the spread of malignant cells from lumps or extra nodal sites. Hence, targeting ACKR3-mediated tumor dissemination is appealing. Our data showing the involvement of the LTB4/BLT_1_R axis in VAL cell migration, could open a way for the development of novel therapeutic approaches in the treatment of DLBCL, potentially in combination with inhibitors of ACKR3 (69–72).

## Methods and materials

### Cell lines and cultures

VAL and DOHH2 cells were cultured in RPMI-1640 supplemented with 10% heat-inactivated Fetal Bovine Serum (FBS), 1% Penicillin/Streptomycin (P/S), 1% GlutaMAX. 1% NEAA, 1% Sodium-Pyruvate and 50 µM β-mercaptoethanol all from Thermo Fisher. Primary murine lymphatic endothelial cells (mLEC) were isolated from lymph nodes of C57BL/6 mice and cultured in αMEM (Thermo Fisher) supplemented with 10% heat-inactivated FBS, 1% P/S. Cells were cultured in humidified air at 37° C with 5% CO_2_.

### mLEC isolation

Freshly harvested lymph nodes were harvested from C57BL/6 mice, and incubated in digestion mixture containing RPMI-1640, 0.25 mg/mL Liberase TL (Roche, Cat: 5401020001), 200 Kunits/mL DNAse I (Sigma-Aldrich, Cat: 10104159001) at 37° C for 1h with occasional shaking. Single cell suspensions were strained using a 70 µm cell strainer (Corning), centrifuged and resuspended in αMEM. Cells were plated on 6-well plates coated with 10 µg/mL PureCol (Collagen, Sigma-Aldrich) and human 10 µg/mL plasma Fibronectin (Sigma-Aldrich). After 3 hours non-adhering cells were removed. 5-7 days later, when >80% confluence was achieved, cells were detached using Accutase (Biological Industries). Under these conditions blood endothelial cells die and the remaining cells are composed of fibroblastic reticular cells (FRCs) (~40%) and LECs. LECs were purified by staining with APC-conjugated anti-mouse CD31 (Biolegend, clone 390) and positively selected using anti-APC microbeads according to manufacturer’s instructions (Miltenyi Biotec).

### Reconstitution of Val VAL-ko cells with ACKR3 variants

ACKR3 and the C-terminus deficient variant ACKR3-ΔC (6) were cloned into CDH-IF1-MCS-COP-GFP vector and nucleofected (Lonza) into CFP^+^ VAL-ko (32) giving rise to VAL-wt rescue and VAL-ACKR3-ΔC. The transfected constructs expressed GFP after a T2A cleavage sequence, which was used for clonal selection by flow cytometry and single cell sorting (FACS-ARIA).

### CRISPR/Cas9 editing of BLT_1_R gene on VAL-wt cells

CRISPR/Cas-9 ribonucleoproteins (crRNPs) targeting *blt_1_r* gene were delivered to VAL-wt cells by transfection with NEON nucleofector (Invitrogen, MA, USA) at 1’600 V, 10 ms, 3 pulses, using the provided buffer R. 10^6^ cells were used with the 10-μl tip transfection. The crRNAs (/AltR1/rCrArU rGrArG rUrCrU rArGrA rCrCrG rCrUrC rArCrG rUrUrU rUrArG rArGrC rUrArU rGrCrU/AltR2/”), tracrRNAs (IDT, USA) and HiFi Cas9 Nuclease V3 (IDT, USA) transfection was performed as indicated in the manufacturer’s instruction (IDT, USA). Deletion of *blt_1_r* was confirmed by genomic sequencing of the locus.

### Flow cytometry analysis and cell sorting

For flow cytometry cells were suspended in MACS buffer (phosphate buffered saline (PBS), 2% FBS, 2mM EDTA) and stained for 15 minutes on ice with the indicated antibodies. For chemokine uptake, 50 nM of fluorescently labelled chemokine (42, 73) was added to cells for 45 min at 37°C. Mean fluorescence intensity (MFI) of the fluorophore were determined by flow cytometry (Fortessa or FACSCanto (BD) and FlowJo software.

### Transwell migration assay

Lymphoma cell migration was measured in transmigration assays using 5 µm transwell inserts. mLECs were seeded on polycarbonate inserts coated with 10 µg/mL fibronectin and 10 µg/mL collagen. Monolayers of endothelial cells were grown by adding medium to the upper compartment, leaving the lower compartment empty.

For LTB4-induced migration lymphoma cells were embedded in a collagen matrix formed by 1.6 mg/mL PureCol, 0.36% PBS supplemented with 0.36% FBS, 0.036% P/S, 1.5 µg/mL recombinant human ICAM-1/CD54 Fc chimera (R&D systems) at 4° C. To induce collagen polymerization the temperature was slowly raised over 45 min to 37°C. Then medium was added on the upper compartment of the transwell inserts for 24 h before the experiment. When indicated, embedded cells were pretreated with 1 µM BIIL315 (OpnMe-Boehringer Ingelheim) for one hour at 37°C. The medium of the upper compartment was then replaced with fresh medium containing 1 µM BIIL315, which was also present in the lower compartment during the experiment.

Lymphoma cells were pretreated with 10 µM AMD3100 (Sigma-Aldrich) for one hour at 37° C and transwell migration was performed in the presence of the same concentration of the inhibitor in the upper and lower compartment. Cells were treated with 2 µg/mL pertussis toxin (PTX) (Sigma-Aldrich) for two hours at 37°C., washed and added to inserts coated with endothelial cells without toxin.

Migration was stimulated with chemoattractants at the indicated concentrations in the lower compartment. Cells were allowed to migrate to the lower chamber for 8 hours. After a brief centrifugation (1 min, 250 x g) of the inserts, cells were collected by centrifugation and resuspended in 100 µL of MACS buffer. For cell number determination cells were counted for 50 sec by flow cytometry.

### LTB4 determination

Cells were resuspended at 10^6^ cells mL^-1^ in PBS and incubated for 30 minutes on ice. Cells were centrifuged at 350g for 3 minutes, re-suspended at 10^7^ cells mL^-1^ in plain RPMI and incubated for 30 minutes at 37°C. Secretion of LTB4 was stopped by centrifugation at 4° C (350g for 3 minutes) and supernatants collected. LTB4 ELISA was performed as indicated in the manufacturer’s instruction (Enzo Life Sciences).

### 3D migration IBDI chambers

Lymphoma cell migration in 3D chemotaxis were performed using µ-Slide Chemotaxis (Ibidi). Cells were embedded in a collagen matrix as described above. To optimize and standardize the architecture of the collagen fibers we slowly induced polymerization by precooling and extending the warming rate from 4°C (liquid) to 37°C (solid) of the collagen matrix (74) (75). The IBD chambers were loaded at 4°C and slowly warmed up to room temperature (20 min) before placing into a 37°C cell incubator. Total warming time to 37°C was about 40 min. The condition allows the formation of thin fibers with homogenous diameter, length and density and prevents the formation of fibers in hierarchical structures (74) (75). After polymerization complete medium (65 µL) was added on both side-reservoirs. Cells embedded in the collagen matrix in the µ-Slide chamber were incubated in a humidified environment at 37°C and 5% CO_2_ for one day. After 24 hours 15 µL of 400 nM CXCL12 were added to one of the reservoirs and migration observed for 6 hours by time-lapse video microscopy (ImageXpress^®^ Molecular Devices) at 2 minutes or 20 seconds time intervals between frames.

### Mice and mouse model

The localized mouse xenograft model was described previously (32). 8–10-week-old mice were injected subcutaneously with 10^7^ VAL or DOHH2 cells in PBS, or only PBS for control groups. All *in vivo* experiments were performed following the rules of the Swiss Federal Veterinary Office guidelines and authorized by the Animal Studies Committee of Cantonal Veterinary.

### Tumor and lymph node processing

A day before culling 50 µL of Evans Blue (2 µg/mL) were injected subcutaneously on the flank close to the xenograft do visualize lymphatic vessels. Localized tumors and draining axillary lymph node were surgically removed and disintegrated with 150 µm Sefar Nitex filters and cells processed for flow cytometry analysis as described (76). Cells of the disintegrated lymph node were resuspended in 150 µL of MACS buffer and analyzed by flow cytometry.

### Immunofluorescence microscopy

Tumors and lymph nodes were collected and fixed with a solution of 4% paraformaldehyde and washed with PBS, 1% FBS and 0.05% NaN_3_. The organs were embedded in low melting agarose (Sigma-Aldrich) and 50-100 µm sections were prepared with a Leica VT1200S Vibratome. Images were acquired with a confocal microscope (Leica SP5) and analyzed with Imaris software (Bitplane).

### RNA-seq

For each condition, five biological replicates of cells were separately cultured, treated and sorted. DLBCL VAL cells were stained with 5 µg/mL anti-human ACKR3 (mAb 11G8). at 4°C for 15 min. Cells were washed twice with MACS Buffer and sorted (5x10^5^ cells) for ACKR3 positivity or negativity in 500 µL of RNA-later (Sigma-Aldrich). RNA-later lysates were centrifuged at 2500g x 5 minutes. The Zymo column RNA Isolation kit was used for RNA extraction (Zymo). Transcripts were processed for RNAseq with E7765L NEBNext Ultra II Directional RNA Library Prep, Sample Purification Beads and E7490L NEBNext^®^ Poly(A) mRNA Magnetic Isolation Module. Poly-A RNA sequencing was performed with Illumina NextSeq500. RNA quantification was performed using Salmon v1.4.0 with hg38 transcriptome reference (77). The expression levels of the transcripts were then compared in the R package DESeq2 v1.28.0 (78).

## Data availability statement

The datasets presented in this study can be found in online repositories. The names of the repository/repositories and accession number(s) can be found here https://www.ncbi.nlm.nih.gov/geo/query/acc.cgi?acc=GSE169144.

## Ethics statement

The animal study was reviewed and approved by Swiss Federal Veterinary Office guidelines and authorized by the Animal Studies Committee of Cantonal Veterinary.

## Author contributions

Conceptualization: PA, MT. Methodology: PA, DP, MT. Investigation: PA, SM, ER, ST. Formal Analysis: MF. Visualization: PA, MT. Supervision: MT. Writing - original draft: PA, DP. Writing - review & editing: PA, MT. All authors contributed to the article and approved the submitted version.
