# Supplementary material for: ACKR3 promotes CXCL12/CXCR4-mediated cell-to-cell-induced lymphoma migration through LTB4 production
Source: Front Immunol. 2023 Jan 12;13:1067885. doi: 10.3389/fimmu.2022.1067885 (PMC9878562; doi:10.3389/fimmu.2022.1067885)
Supplement: Supplementary file 1 [file DataSheet_1.pdf]

Supplementary figure 1

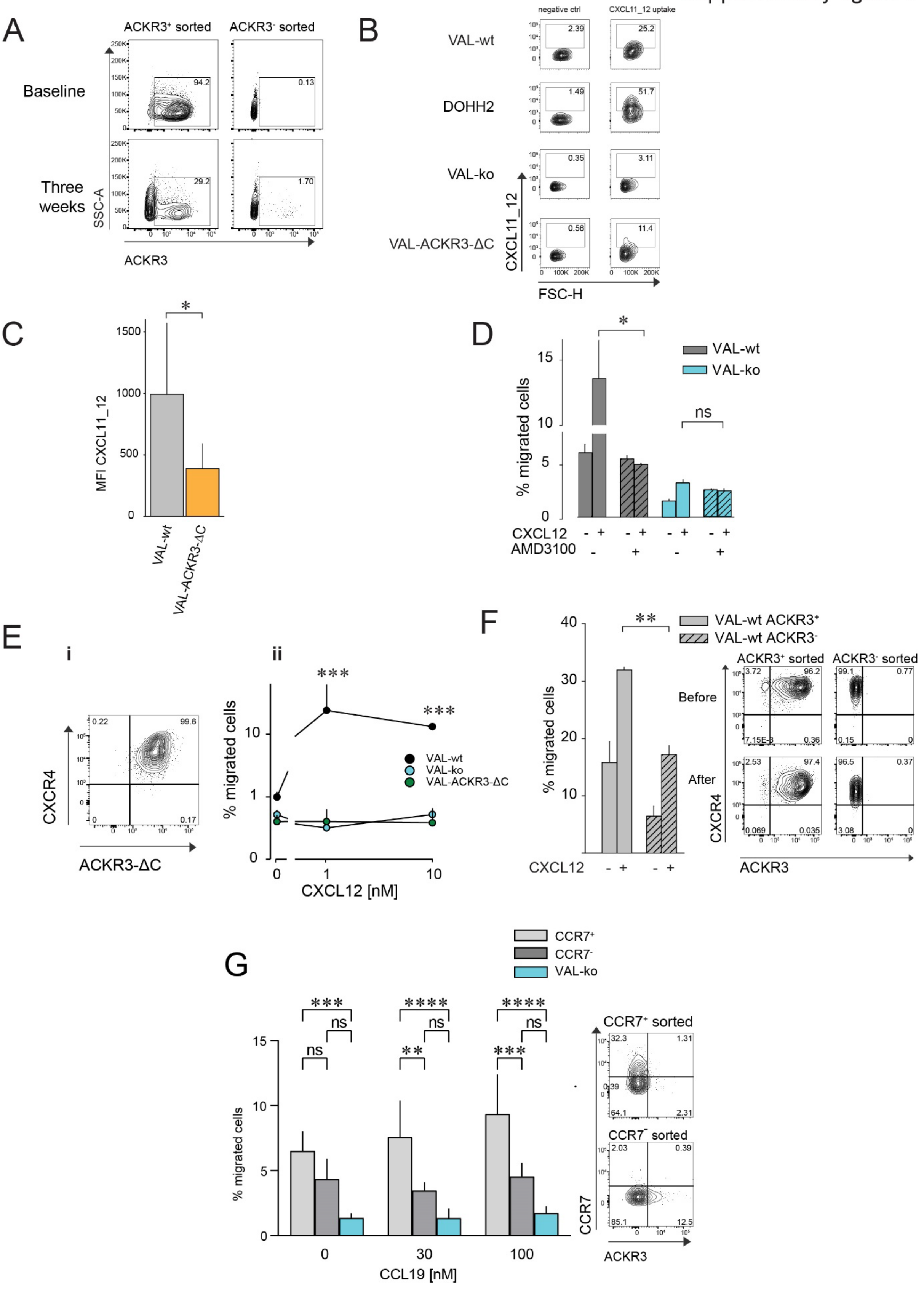

Supplementary Fig. S1: (A) ACKR3 (mAb 11G8) surface expression on sorted VAL cells was measured by flow cytometry. ACKR3 surface expression of $ACKR3^{+}$ (left) and $ACKR3^{-}$ (right) VAL-wt at day 0 of sorting (upper panels, baseline) and after three weeks of culture (lower panels). **(B)** Flow cytometry plots showing gate strategy of CXCL11_12 uptake by DLBCL cells. Controls without addition of CXCL11_12 AF647 (left panels) and (right panels) uptake of CXCL11_12 AF647. Representative plots from three independent determinations. **(C)** Uptake of the chimeric chemokine CXCL11_12 AF647 by VAL-wt and VAL-ACKR3-ΔC. Uptake performed as described in Fig 1B. MFI reports the mean ± SD. Cumulative data from two independent experiments measured in triplicates (means ± SD, Turkey's multiple comparison ONE-WAY ANOVA, *p≤0.02). **(D)** Transendothelial migration of lymphoma cells. VAL cell migration through mLEC was measured as described in Fig. 1C. Migration of VAL-wt and VAL-ko stimulated with 10 nM CXCL12 was measured in the absence (plain bars) and presence (hatched bars) of 10μM of the CXCR4 antagonist AMD3100. Cell migration was calculated as percentage of the input (% migrated cells). Representative example of two independent experiments measured in triplicates (means ± SD, Holm-Sidak method, ONE WAY ANOVA, ns p≥0.05, * p≤0.05). **(E)** **(i)** ACKR3 (mAb 11G8) and CXCR4 (mAb 12G5) surface expression on VAL-ACKR3-ΔC was measured by flow cytometry. Representative plot of three independent determinations. **(ii)** Transendothelial chemotaxis through mLEC of VAL cells induced by the indicated concentrations of CXCL12. The % migrated cells was calculated as in Fig. 1C from input of VAL-wt (black symbols), VAL-ko (cyan symbols) and VAL-ACKR3-ΔC (green symbols). Representative data of three independent experiments measured in triplicates (means ± SD, Holm-Sidak method, ONE WAY ANOVA, ***p≤0.001). **(F)** Left panel: transendothelial migration of sorted VAL cells stimulated with 10 nM of CXCL12. Right panels: flow cytometry plots showing ACKR3 (mAb 11G8) and CXCR4 (mAb 12G5) surface expression on VAL-wt $ACKR3^{+}$ (left) and $ACKR3^{-}$ (right) sorted cells, before (upper panels, before) and after migration (lower panels, after). Cell migration was calculated as described above. Representative example of two independent experiments measured in triplicates (means ± SD, Student's t-test **p≤0.01). **(G)** Left panel: transendothelial chemotaxis of VAL

cells in the presence of increasing concentrations of CCL19 (30, 100, nM). The % migrated cells from input of CCR7$^+$ (light grey bars), CCR7$^-$ (dark grey bars) sorted and VAL-ko (cyan bars). Cumulative data of three independent experiments performed in triplicates (means ± SD, Turkey's test, ONE-WAY ANOVA, ns, $p \geq 0.05$, **$p \leq 0.01$, ***$p \leq 0.001$, ****$p \leq 0.0001$). Right panels: flow cytometry plots showing CCR7 (mAb G043H7) surface expression on VAL-wt CCR7$^+$ (upper) and CCR7$^-$ (lower) sorted cells used in the transmigration experiment (left panel).

Supplementary figure 2

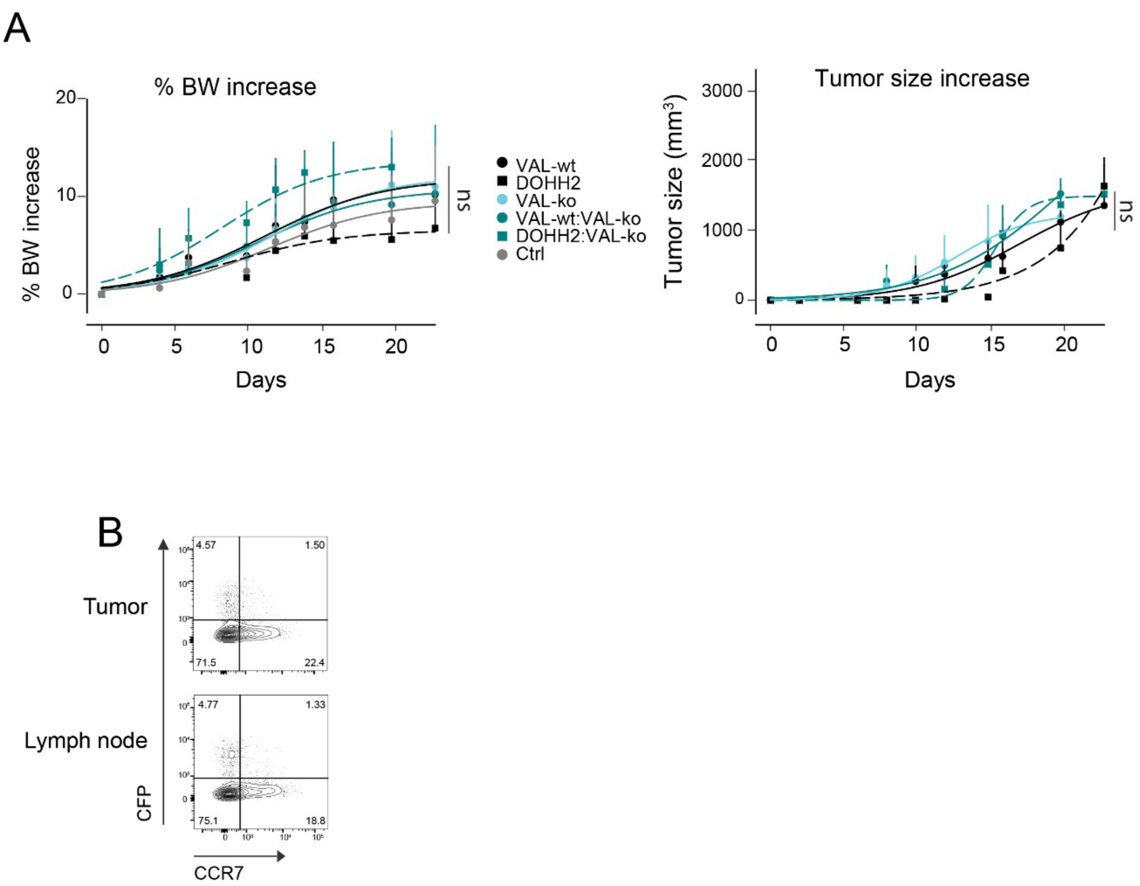

Supplementary Fig. S2: (A) Body weight (BW) increase (left) and tumor volume (right) increase of NOD/SCID common γ-chain^{ko} mice over time. BW was calculated with the formula (BW_n-BW_0/BW_0)*100. Tumor volumes measured with a caliper were calculated with the formula: (Depth)^2*Lenght/0.4. Body weight and tumor volumes statistics are reported as means ±SD, Shapiro-Wilk test: ns $p \geq 0.05$. **(B)** Surface expression of CCR7 on VAL cells and CFP expression in VAL-ko extracted from a representative localized tumor (upper panel) and from the corresponding lymph node (lower panel). Representative data from one experiment shown in A (n= 4-5).

Supplementary figure 3

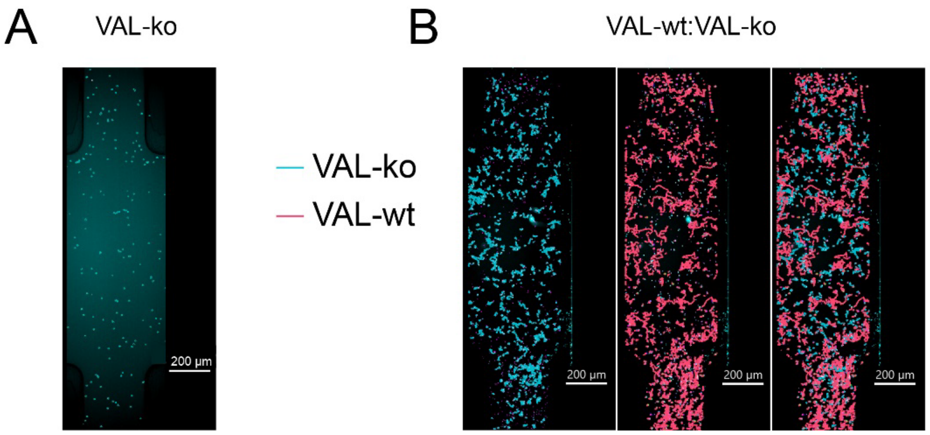

Supplementary Fig. S3: (A) Snapshot a 3D migration chamber, tracks of VAL-ko cells are shown in cyan color. Tracking analyses were performed using Imaris software on a video of 6 hours. **(B)** Snapshot of a 3D migration chamber showing full-tracks of VAL-ko (left, cyan tracks) and VAL-wt (center, red tracks) mixed in a 1:1 ratio. Both tracks are merged on the right panel. Tracking analyses were performed using Imaris software on a video of 6 hours.

Supplementary figure 4

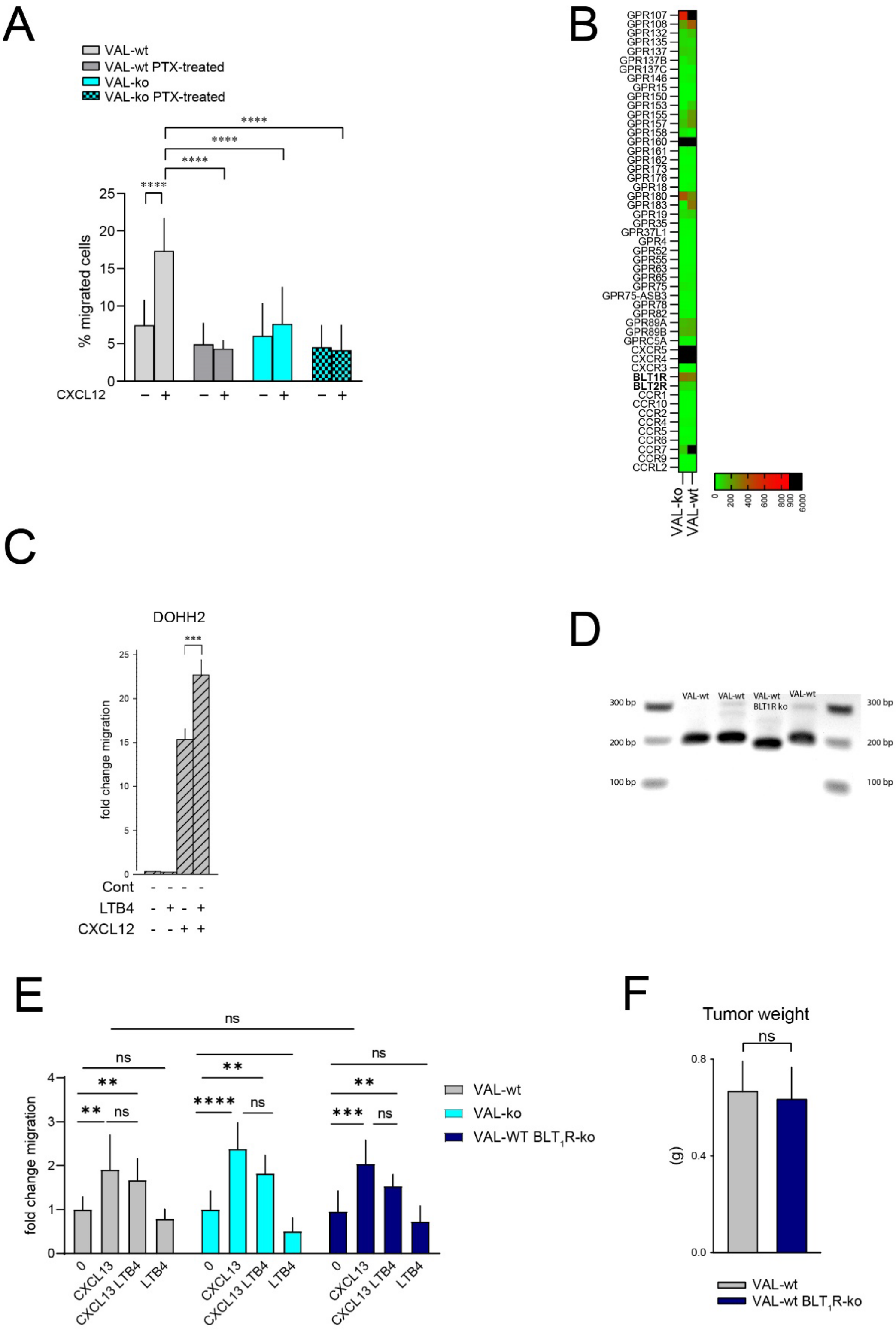

Supplementary Fig. S4: (A) Transmigration was performed and calculated as described in Fig. 1C. Migration towards 10 nM CXCL12 of VAL cells was measured in the absence and presence of 2 µg/mL pertussis toxin, PTX (see methods). VAL-wt untreated (light grey) or treated with PTX (dark grey) and VAL-ko untreated (cyan) or treated with PTX (cyan, black checkers). Cumulative data of three independent experiments performed in triplicates (means ± SD, Turkey's multiple comparisons, ONE WAY ANOVA, ****p≤0.0001). **(B)** Heat map of GPCRs transcripts expressed in VAL-ko (left) and VAL-wt (right) cells. RNA-seq performed in biological pentaplicates. **(C)** DOHH2 cells were embedded in a collagen matrix on the transwell insert. Fold change of DOHH2 (light grey, hatched bars) migrating in response to 10nM LTB4 and 10 nM CXCL12. Representative data from three experiments performed in quadruplicates (means ± SD, Turkey's multiple comparisons, ONE WAY ANOVA, ***p≤0.001). **(D)** Image showing amplicon sizes of VAL-wt clones and VAL-wt BLT_1R ko, on agarose gel (1%). Acquisition performed with UV transilluminator. **(E)** VAL-wt, VAL-ko and VAL with deleted blt_1r were embedded in a collagen matrix on the transwell insert and allowed to migrate towards 200 nM CXCL13 or 10nM LTB4 or 10nM LTB4 and 200nM CXCL13 for 6h as in Fig. 6B. Pooled data from three independent experiments performed in triplicartes. Unpaired t-test **p≤0.01, ***p≤0.001and **** p≤0.0001. **(F)** Tumor weights from NOD/SCID common γ-chain^{ko} mice injected with 10^7 VAL-wt (grey bar) and VAL-wt BLT_1R ko (blue bar). Weights are reported as means ±SD from two independent xenograft experiments with 4-5 animals per group. ns, p≥0.05.

Supplementary movies S1-3: (Movie_1) Time-lapse video showing the track of a VAL-ko (red circle 3) and the movement of a VAL-wt (red circle 2) migrating to a position where a VAL-wt cell was located and left (red circle 1). **(Movie_2)** Time-lapse video showing the tracks of three VAL-ko cells (red circles 4-5-6) migrating to a position where two VAL-wt cells were located (red circles 1-2). **(Movie_3)** Time-lapse video showing the tracks of two VAL-wt cells (red circles 2-3) migrating to a position where a VAL-wt cell was located (red circle 1).
